# Testing a Behavioral Activation Gaming App for Depression During Pregnancy: Multimethod Pilot Study

**DOI:** 10.2196/44029

**Published:** 2024-01-26

**Authors:** Rachel C Vanderkruik, Craig Ferguson, Lauren A Kobylski, Joseph J Locascio, Gabriella E Hamlett, Parker C Killenberg, Robert Lewis, Noah Jones, Ella T Rossa, Hannah Dineen, Rosalind Picard, Lee S Cohen

**Affiliations:** 1 Center for Women's Mental Health Massachusetts General Hospital Boston, MA United States; 2 Department of Psychiatry, Harvard Medical School Boston, MA United States; 3 MIT Media Lab Massachusetts Institute of Technology Cambridge, MA United States; 4 Department of Psychological & Brain Sciences George Washington University Washington, DC United States; 5 Department of Neurology, Harvard Medical School Boston, MA United States; 6 Department of Psychology, Harvard University Cambridge, MA United States

**Keywords:** perinatal depression, pregnancy, behavioral activation, mobile app, digital intervention, mobile phone

## Abstract

**Background:**

Depression during pregnancy is increasingly recognized as a worldwide public health problem. If untreated, there can be detrimental outcomes for the mother and child. Anxiety is also often comorbid with depression. Although effective treatments exist, most women do not receive treatment. Technology is a mechanism to increase access to and engagement in mental health services.

**Objective:**

*The Guardians* is a mobile app, grounded in behavioral activation principles, which seeks to leverage mobile game mechanics and in-game rewards to encourage user engagement. This study seeks to assess app satisfaction and engagement and to explore changes in clinical symptoms of depression and anxiety among a sample of pregnant women with elevated depressive symptoms.

**Methods:**

This multimethod pilot test consisted of a single-arm, proof-of-concept trial to examine the feasibility and acceptability of *The Guardian*s among a pregnant sample with depression (N=18). Participation included two web-based study visits: (1) a baseline assessment to collect demographic and obstetric information and to assess clinical symptoms and (2) an exit interview to administer follow-up measures and explore user experience. Participants completed biweekly questionnaires (ie, Patient Health Questionnaire-9 and Generalized Anxiety Disorder-7) during the trial to assess depression and anxiety symptom severity. App satisfaction was measured using 2 self-report scales (ie, Mobile Application Rating Scale and Player Experience of Needs Satisfaction scale). Engagement with *The Guardians* was captured using game interaction metric data. We used backward-eliminated mixed effects longitudinal models to examine the effects of app engagement and satisfaction and length of time in the study on symptoms of depression and anxiety. Content analysis was conducted on qualitative data from exit interviews.

**Results:**

The 15-day and 30-day overall app retention rates were 26.6% and 15.1%, respectively. Mixed effects models found significant negative main effects of *week in study* (β=−.35; *t*_61_=−3.05; *P*=.003), number of *activities completed* (β=−.12; *t*_61_=−2.05; *P*=.04), *days played* (β=−.12; *t*_58_=−2.9; *P*=.005), and satisfaction, according to the Mobile Application Rating Scale (β=−3.05; *t*_45_=−2.19; *P*=.03) on depressive symptoms. We have reported about similar analyses for anxiety. There is preliminary evidence suggesting *harder* activities are associated with greater *mood improvement* than *easier* activities. Qualitative content analysis resulted in feedback falling under the following themes: activities, app design, engagement, fit of the app with lifestyle, perceived impact of the app on mood, and suggestions for app modifications.

**Conclusions:**

Preliminary results from this multimethod study of *The Guardians* indicate feasibility and acceptability among pregnant women with depression. Retention and engagement levels were more than double those of previous public mental health apps, and use of the app was associated with significant decrease in depressive symptom scores over the 10-week trial. *The Guardians* shows promise as an effective and scalable digital intervention to support women experiencing depression.

## Introduction

### Background

There is growing recognition of the burden of depression during the perinatal period (ie, pregnancy and postpartum periods). Prevalence estimates of perinatal depression (PD) range between 10% and 15% in high-income countries and are even higher in low- and middle-income countries [1,2]. If left untreated, there can be significant and detrimental consequences for both the mother and infant [3,4]. In response to this public health problem, the US Preventive Services Task Force recently recommended that clinicians provide or refer pregnant and postpartum individuals who are at increased risk of PD to counseling interventions [5]. Pharmacologic and nonpharmacologic treatments to manage symptoms of depression during pregnancy are available, yet most women do not receive the treatment they need [6]. Furthermore, targeting depression specifically during *pregnancy* is critical in addressing PD, as depression during pregnancy is a key risk factor for postpartum depression [7], and thus, reducing depression during pregnancy can reduce the risk for postpartum depression. Given the number of individuals affected by PD and significant barriers to care [8,9], strategies that provide increased access to evidence-based treatment approaches are needed.

There is evidence suggesting that women may prefer psychotherapeutic approaches over antidepressant medications during pregnancy [10]. Behavioral activation (BA) is one such psychotherapeutic approach based on the theory that, as individuals become depressed, they tend to engage in avoidance and isolation, which then maintains or worsens their depressive symptoms. BA encourages the individual to gradually increase engagement in activities that serve as “behavioral antidepressants” and decrease their avoidance and isolation [11]. Several meta-analyses support the effectiveness of BA [12,13], and it has been found to be comparable in efficacy with antidepressant medications among adults with major depression [14]. BA has been considered to be advantageous compared with other treatments given the simplicity of the intervention, strong retention rates, and enduring effects over a 2-year follow-up, without the concern of side effects associated with some medications [15,16]. There is emerging evidence supporting BA, specifically among pregnant individuals, with findings that BA can offer significant depression-related, anxiety-related, and stress-related benefits for this population [17].

There is support for scalability and even global dissemination of BA given how it can be delivered by nonspecialists, is cost-effective, and has demonstrated cross-cultural fit and adaptability [18]. Furthermore, BA has been identified as being suitable for “computer-based interventions that would involve no therapist input beyond an initial assessment,” which could dramatically improve the accessibility of effective treatments for depression [15]. Digital technology, including mobile health apps, has been cited as a promising strategy for increasing access to evidence-based interventions for mental health for several reasons, including constant availability, equity of availability, immediate support, low costs, lack of geographic barriers, and reduced need for direct mental health service provision (particularly given the shortage of clinicians in certain locations) [19]. A systematic review of the effectiveness of mobile apps for monitoring and managing mental health symptoms or disorders concluded that there is support for the potential of mobile apps to effectively reduce the burden of mental health symptoms; yet, further robust studies are needed to develop and test evidence-based apps [20]. Therefore, a digital BA app could be a promising way to increase access to support for depression among vulnerable or underrepresented populations.

It is, thus, not surprising that there have been recent efforts to develop BA apps, including a self-help brief BA app focused on activity scheduling for the general population called Moodivate. A preliminary randomized controlled trial (RCT) recruited adults with elevated depression from primary care practices (N=52) and randomized them to receive (1) Moodivate, (2) an active control cognitive behavioral therapy–based mobile app (MoodKit), or (3) treatment as usual (no app) [21]. Study retention was relatively high, with approximately 70% of Moodivate participants continuing to use the app 1 month after trial enrollment and 50% at the end of the 8-week study period. Compared with treatment as usual, participants in both app conditions experienced significant reductions in depressive symptoms over time, and these treatment gains were sustained throughout the trial period. These preliminary results support the potential feasibility of a BA app, such as Moodivate, as a treatment for depression. Other preliminary studies have exhibited promising findings regarding the BA apps’ abilities to provide motivation for adults with depression to plan enjoyable activities [22] and acquire insight into their own behavior and impact on mood [23]. However, these BA apps are not widely and freely available to the public, and more rigorous studies are needed regarding their impact on depressive symptoms and among certain populations.

A recent meta-analysis provides support for the efficacy and acceptability of internet-delivered interventions for pregnant women and highlights the opportunity to leverage technology for interventions targeting this population, including for mental health symptoms during pregnancy [24]. Given the challenges of engaging pregnant people in treatment, preferences for depression treatment in nonspecialty settings, and limited availability of services targeting depression during pregnancy [25], a widely available and engaging BA app could be a highly impactful service for individuals with PD. Although a variety of apps designed to improve health and well-being have been developed, most are not grounded in evidence-based principles, and most struggle to keep users engaged [26-28]. Many such apps used typical “gamification” techniques (ie, awarding users with badges or points for using the app), which are designed to increase adherence but often fail to give the user reasons to care about those rewards. Thus, long-term retention and engagement in the app may be affected [29]. A different approach used by a mobile game called *The Guardians: Unite the Realms* provides immediate rewards for using BA therapeutic techniques as part of the app and gives those rewards inherent value and meaning through their use within the game’s mechanics [30,31].

*The Guardians: Unite the Realms* is a novel gaming BA app that is free to be downloaded by the general public with iPhone Operating System and Android. The app was developed by an author of this paper (CF) with input from experts in digitizing therapies to ensure that it maintains the core qualities and effectiveness of BA. *The Guardians* was designed to increase adherence to app-delivered BA by embedding BA techniques into the unique context of a mobile game, giving intrinsic incentives for users to continue using the app [30]. Since its launch in April 2020, *The Guardians* has collected anonymized gameplay data from about >12,500 users and saved them to its secure backend database for research and gameplay recovery purposes, as specified in the app’s privacy policy [29]. The 15-day and 30-day overall retention rates of 10% and 6.6%, respectively, are more than double the average retention rates for mental health apps of 3.9% (IQR 10.3%) and 3.3% (IQR 6.2%), as reported by Baumel et al [27] and Ferguson et al [30]. Furthermore, the 1-day and 28-day observed retentions of 37.9% and 7.3%, respectively, suggest that *The Guardians* has retention rates that are comparable with those of the top 15% of mobile games. Although there has not yet been a formal study of how the use of *The Guardians* app may influence depressive symptoms, >80% of the BA *activities completed* as part of the app resulted in the user feeling at least “a little bit better” [30]. *The Guardians* is a widely available BA gaming app with a novel approach to engage users, and preliminary data show a positive impact on mood among its users.

### This Study

To the best of our knowledge, there are no gaming BA apps that have been developed specifically for a pregnant population with elevated depressive symptoms. Given the promising preliminary data about *The* Guardians in terms of engagement metrics and user-reported improvement in mood from app-related activities, along with the need for more novel approaches to address depressive symptoms during pregnancy, this pilot study sought to assess app engagement and to explore the potential resulting changes in mental health symptoms among a pregnant sample with elevated depressive symptoms. As anxiety is often comorbid with depression during pregnancy [32] and there is some preliminary evidence suggesting that BA may also be beneficial for anxiety symptoms, we also sought to explore the changes in anxiety symptoms among users of *The Guardians* app [33,34].

Furthermore, we sought to capture feedback from study participants about how a future version of *The Guardians* could be specifically tailored for a pregnant population, as we used the publicly available app geared for a general population in this pilot study. Incorporating a user-centered design into app development is linked to high usability, low risk of failure, reduced costs, and high overall quality [35] and can help inform app design and implementation according to feedback about the target users’ needs [36,37]. Thus, the aims of this pilot study were to explore engagement with and impact of *The Guardians* among pregnant individuals with elevated depressive symptoms, while also qualitatively exploring user experience and gathering suggestions for a future iteration of the app that could be tailored specifically for the context of pregnancy.

## Methods

### Participants and Recruitment

Consistent with a pragmatic trial, the inclusion criteria for this single-arm pilot study were minimal. Women were eligible to participate if they were pregnant, aged >18 years, and English speaking; had access to a smartphone; and had a Patient Health Questionnaire-9 (PHQ-9) score of at least 10 (indicative of a possible depressive episode). Exclusion criteria included a diagnosis of bipolar or psychotic disorder, active mania, psychosis, substance abuse, or immediate risk of self-harm based on PHQ-9 responses and clinician judgment. For this initial pilot study, we focused on pregnancy and excluded women in the postpartum period as there are other complicating factors in the postpartum period (eg, sleep disruption and demands of caring for a newborn) that can affect the ability to engage in the app and completion of app activities. As this was a completely web-based study, participants could be located anywhere in the United States. Participants were recruited through social media advertising, clinician referrals, and the Massachusetts General Hospital Center for Women’s Mental Health website [38]. Individuals interested in participating were instructed to reach out via mobile phone or email to the study’s research assistant using the contact information provided in the study advertisements.

### Procedures

Following an eligibility screening via mobile phone, participants provided verbal informed consent and completed a baseline assessment with a trained research assistant. During the baseline interview, demographic variables, pregnancy characteristics, and psychiatric history (ie, diagnosis and treatment) were collected. At the conclusion of the baseline assessment, the research assistant instructed the eligible participants about how to download *The Guardians* app onto their personal smartphone. To approximate real-world mobile app use, there were no further app-related engagement prompts from study staff after the baseline assessment.

During the 10-week study period, participants were invited to complete web-based biweekly surveys via REDCap (Research Electronic Data Capture; Vanderbilt University) [39,40] to assess their depressive and anxiety symptoms (refer to details in the *Measures* section). If participants did not complete these biweekly assessments, a research assistant contacted the participant to remind them to complete the survey. A final assessment was conducted over mobile phone at the end of the 10-week trial to administer follow-up measures and conduct a brief qualitative exit interview. In addition to assessments via REDCap surveys, app analytics (eg, days played and activities completed) were captured through *The Guardians* via the game’s cloud save and gameplay recovery functionality. The participant data were compared with the anonymized gameplay data gathered from the app’s public users. All data gathered by *The Guardians* were anonymized and collected for research purposes, as stated in the app’s privacy policy [30].

### Ethical Considerations

All the study procedures were approved by the Mass General Brigham institutional review board (protocol number 2021P001400). All participants provided informed consent, and all study data were deidentified before analysis. Participants were not provided compensation for their participation in the study.

### Overview of The Guardians: Unite the Realm App

As noted previously, *The Guardians: Unite the Realms* is a mobile game designed to increase adherence to a modified BA therapy. A detailed description of the game has been published previously [30]. In brief, *The Guardians* is divided into 3 realms, each of which unlocks automatically after 28 days, regardless of game progression. In each realm, the user is asked to defeat an enemy character by collecting pets and sending them on missions. The pets automatically complete each mission after 10 to 60 seconds in real time. Once the pets complete a mission, they are given experience points and other rewards that can be used to further the in-game progress. Players are given a limited amount of regenerable “stamina,” which they must spend to send pets on missions. Thus, the challenge comes from carefully managing which pets should go on which missions based on the resources and stamina currently available. Players cannot lose the game or undo any progress. Every day, the player is prompted through the app to complete activities that will reward them with more pets. Players are notified to complete their daily activity in the game and via mobile phone notifications.

The player is prompted to either pick from a list of 75 suggested real-world activities or to choose their own activity. The preselected activities are divided into 3 effort levels (low, medium, and high) and into 5 categories (basics, arts and crafts, social, fitness, and fun); in-game rewards are the same regardless of effort level and category. Once the player chooses their activity for the day, the game instructs the participant to log the activity in the app once it is complete. Players cannot log the activity until sufficient time to complete their activity has passed. After logging an activity, players are prompted to reflect about how they feel after completing it, rating their *post activity mood improvement* on a scale of “1: Much worse” to “5: Much better.” Reflecting about how an activity affects mood is a key ingredient from BA therapy that has been integrated into *The Guardians*.

### Measures

#### Diagnostic Assessment

In addition to collecting demographic information, the Mini International Neuropsychiatric Interview [41] was administered at the baseline assessment to evaluate the diagnostic criteria for a current major depressive episode (MDE) and to assess comorbid psychiatric illnesses. It is a structured diagnostic assessment that evaluates the current existence of a variety of psychiatric disorders based on the *Diagnostic and Statistical Manual of Mental Disorders* (Fifth Edition) criteria. To meet the MDE criteria, participants must report having had a depressed mood most of the day or markedly diminished interest or pleasure in activities for a period of at least 2 weeks. They must also endorse at least 5 of the following symptoms: significant unintentional weight or appetite change, insomnia or hypersomnia, psychomotor agitation or retardation, fatigue, feelings of worthlessness or inappropriate or excessive guilt, decreased ability to concentrate or make decisions, and recurrent thoughts of death or suicidal ideation.

#### Primary Outcome: App Satisfaction and Engagement

Overall, 2 self-report measures were used to assess satisfaction with *The Guardians* among this sample at the end of the 10-week trial using the Mobile Application Rating Scale (MARS) and the Player Experience of Needs Satisfaction scale (PENS). MARS is a 23-item self-report survey that contains 4 objective quality scales (engagement, functionality, esthetics, and information quality) and 1 subjective quality scale to classify and assess the quality of mobile health apps [42]. All items are rated on a scale of 1 to 5, and the participants’ score is averaged across all items. High MARS ratings indicate a more usable and high-quality app. PENS is a 6-item survey measuring participants’ play experiences: 3 items measuring competence (eg, “I feel competent at the game”) and 3 items measuring autonomy (eg, “The game lets you do interesting things”) [43,44]. This self-report scale is based on the theory that video games have the potential to satisfy the basic psychological needs for competence, autonomy, and relatedness. Items are rated on a scale of 1 (do not agree) to 5 (strongly agree), and high PENS ratings indicate that the player felt the game met more of their otherwise unmet needs and are associated with high long-term retention [43].

Metrics of engagement captured in the app included *Day-N user retention*, defined as the proportion of users who interact with the game or complete an activity on the Nth day since they installed the game, where day 1 is the first day after installation, and the denominator is the number of users who install the game on day 0 [30,45]. To compare retention rates among participants in this study with those of public users of *The Guardians*, we used data collected from public users (as per the privacy policy [30]) and stored them in *The Guardians* database. Other engagement metrics and data collected in the app included *days played* (ie, the number of days a participant logged into the app) and *activities completed* (ie, the total number of activities that a participant completed in the game). The exit interview, described further in the following sections, additionally captured qualitative feedback about app acceptability, quality, and overall satisfaction.

#### Secondary Outcomes: Changes in Depression and Anxiety Symptom Severity

Depressive symptom severity was assessed using PHQ-9 on a biweekly basis during the 10-week trial using a REDCap survey. PHQ-9 is a well-validated self-report measure consisting of 9 Likert-style items assessing various depressive symptoms [46]. If a participant endorsed suicidality at any point during the trial or via the PHQ-9 suicidality item, further assessment of suicidal ideation and behaviors was performed using the Columbia Suicide Severity Rating Scale [47]. A safety protocol was triggered if the participant endorsed item 9 of PHQ-9 (thoughts of being better off dead) or if the participant endorsed suicidal intent or plan upon completion of the Columbia Suicide Severity Rating Scale. Following a safety protocol trigger, a study clinician would contact the participant as soon as possible and, according to clinical judgment, call the participant’s emergency contact. Participants were also provided with safety resources, including the National Suicide Prevention Hotline, and the safety protocol was modeled after that used in a large federally funded study (Preventing Depressive Relapse in Pregnant Women with Recurrent Depression; National Institute of Mental Health; NCT03623620), where participants (N=500) were assessed for depression across the pregnancy and postpartum periods [48].

Participants also completed the Generalized Anxiety Disorder-7 (GAD-7) scale, a 7-item scale assessing symptoms of anxiety biweekly [49]. As noted in the description of *The Guardians* previously, an additional assessment of *post activity mood improvement* was captured in the app by prompting users to rate how they felt after completing an app-based activity on a scale of “1: Much worse” to “5: Much better.”

#### Qualitative Inquiry: Exit Interview

Participants were invited to complete a brief, 30-minute, web-based exit interview with a research assistant at the end of the 10-week trial. During this interview, participants were asked about perceptions regarding their level of engagement with the app, approach to selecting activities, motivation levels, and aspects of the game that they liked or disliked. Participants were also invited to share their experiences with using the app, perceptions about its impact on their mood, and any recommendations regarding how to best tailor the app for pregnant individuals. Refer to Multimedia Appendix 1 for the exit interview questions.

### Data Analysis

Quantitative data were analyzed using SAS software (version 9.4; SAS Institute). Demographics, user engagement, ratings of satisfaction with the app (eg, activities completed, days played, MARS, and PENS), average *post activity mood improvement*, and depression and anxiety symptoms were analyzed using descriptive statistics. Pearson correlation analyses were conducted to explore the correlation between metrics of app satisfaction (ie, MARS and PENS) and engagement metrics (ie, activities completed and days played). A best-fit curve for the participant app user data was created by assuming an exponential decay in app retention. The user retention must have a day-0 retention of 1 by definition and can be assumed to have an eventual plateau of long-term dedicated users, resulting in the formula, y=(1 − α) e^−λ x^ + α [50]. A series of independent group 2-tailed *t* tests explored the differences in baseline measures of depression (PHQ-9) and anxiety (GAD-7) among participants who completed the final 10-week assessment and those who did not complete this final study assessment. A mixed effects General Linear Model was run with a dependent variable of *post activity mood improvement* rating versus the fixed effect of effort level (ie, easy, medium, and hard). The random term was the participants.

We also conducted analyses to explore how app engagement metrics (ie, activities completed and days played) were associated with symptoms of depression (PHQ-9) and anxiety (GAD-7) over the 10-week trial. To examine the effects of the intervention, as predicted by number of *activities completed* and *days played*, in separate analyses, on symptoms of depression and anxiety, we used backward-eliminated mixed effects longitudinal models for the dependent variables of PHQ-9 and GAD-7, also in separate analyses. The fixed predictors in these models were the numeric variable *week in study* (both linear and quadratic components) and either number of *activities completed* or number of *days played* and their interactions with *week in study*. We included baseline age and gestational age as additional covariates in the models. The random term was participants interacting with linear *week in study*. For all models, model residuals were checked for conformance to model assumptions of normality.

We also used backward-eliminated mixed effects longitudinal models to examine the effect of the overall app satisfaction rating (MARS), needs met through the app (PENS), and a binary predictor indicating whether treatment (ie, psychotropic medication or psychotherapy) was started midstudy for symptoms of depression (PHQ-9) and anxiety (GAD-7), in separate analyses. The random term was participants interacting with time, *week in study*. For all models, we calculated the proportion of variance in the dependent variable accounted for by the fixed effects and by fixed and random variables combined.

Content analysis [51] of the transcribed exit interviews was conducted to identify key themes regarding design features that participants reported as important for the usability and acceptability of the app. A team of 2 coders (LAK and HD) first familiarized themselves with the transcripts, making notes about the themes observed in the qualitative data. Key concepts from the transcripts were used to develop a codebook; codes were developed inductively from the data and deductively from interview topic areas, in addition to exploring the study participants’ perceptions about the design and usability of the app, the app’s impact on mood, and recommendations for the app’s improvement. The coders met to review the codebook and organize codes into broad categories, with guidance from the first author of this paper, who has experience in qualitative methods (RCV). Deidentified transcripts were coded and analyzed using Dedoose (version 9.0.17), a qualitative analysis software program [52].

## Results

### Overview

**Figure 1:**
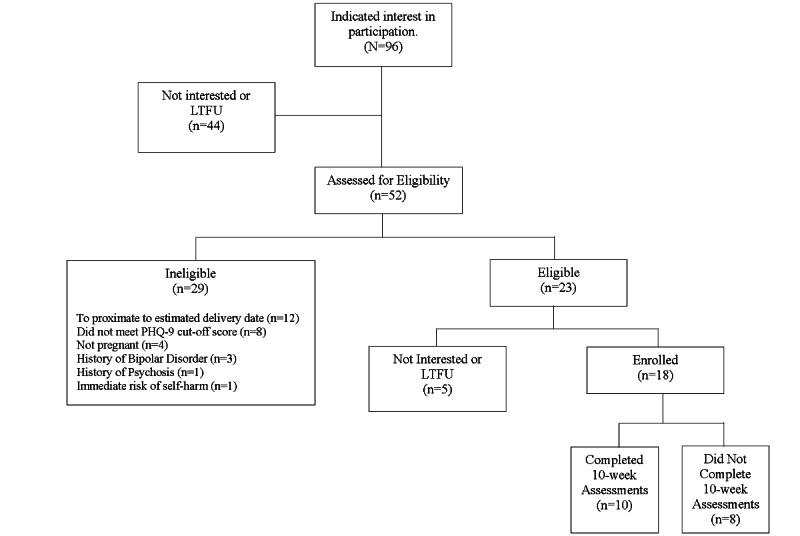
Study participant flow diagram. PHQ-9: Patient Health Questionnaire–9.

During the study enrollment period from September 2021 to April 2022, a total of 96 women indicated interest in participating in this pilot study. Refer to Figure 1 for the flow of participants, including reasons for ineligibility. Ultimately, 24% (23/96) women screened eligible for participation, and 19% (18/96) women enrolled in the study. Of the 18 participants, 10 (56%) completed the final assessment at the end of the 10-week study; we only have data on this 56% (10/18) of the participants for certain measures captured only at the end of the study (eg, PENS, MARS, and exit interview). Table 1 describes the baseline characteristics and demographics of the enrolled sample.

**Table 1:** Participant characteristics at baseline (N=18).

Categories			Values
Age (years), mean (SD)			34.3 (3.0)
Gestational age (weeks), mean (SD)			17.1 (7.1)
**Race and ethnicity, n (%)**			
	Asian	2 (11)	
	Black or African American	4 (22)	
	Hispanic or Latina	2 (11)	
	Non-Hispanic or Latina	16 (89)	
	White	12 (67)	
**Sexual orientation, n (%)**			
	Heterosexual	15 (83)	
	Bisexual	2 (11)	
	Queer	1 (6)	
**Marital status, n (%)**			
	Married	12 (67)	
	Divorced	1 (6)	
	Never married	5 (28)	
**Employment status, n (%)**			
	Employed	16 (89)	
	Student	1 (6)	
	Disabled or unable to work	1 (6)	
**Insurance status, n (%)**			
	Private health insurance	17 (94)	
	Medicaid	1 (6)	
**Education level, n (%)**			
	Postgraduate training	12 (67)	
	Bachelor’s degree	4 (22)	
	Associate degree	1 (6)	
	Some high school	1 (6)	
**Treatment, n (%)**			
	Psychiatric medication in past 2 months	9 (50)	
	Psychosocial treatment in past 2 months	8 (44)	
**Symptom severity (score), mean (SD)**			
	PHQ-9^a^	13.2 (4.7)	
	GAD-7^b^	9.8 (4.2)	
**MINI^c^ diagnoses at baseline, n (%)**			
	Major depressive episode	18 (100)	
	Generalized anxiety disorder	10 (56)	
	Agoraphobia without panic disorder	5 (28)	
	Panic disorder with agoraphobia	1 (6)	
	Social phobia	1 (6)	
	Obsessive-compulsive disorder	1 (6)	
	Posttraumatic stress disorder	1 (6)	
	Drug dependence	1 (6)	

^a^PHQ-9: Patient Health Questionnaire–9.

^b^GAD-7: Generalized Anxiety Disorder–7 item.

^c^MINI: Mini International Neuropsychiatric Interview.

### App Satisfaction and Engagement

**Figure 2:**
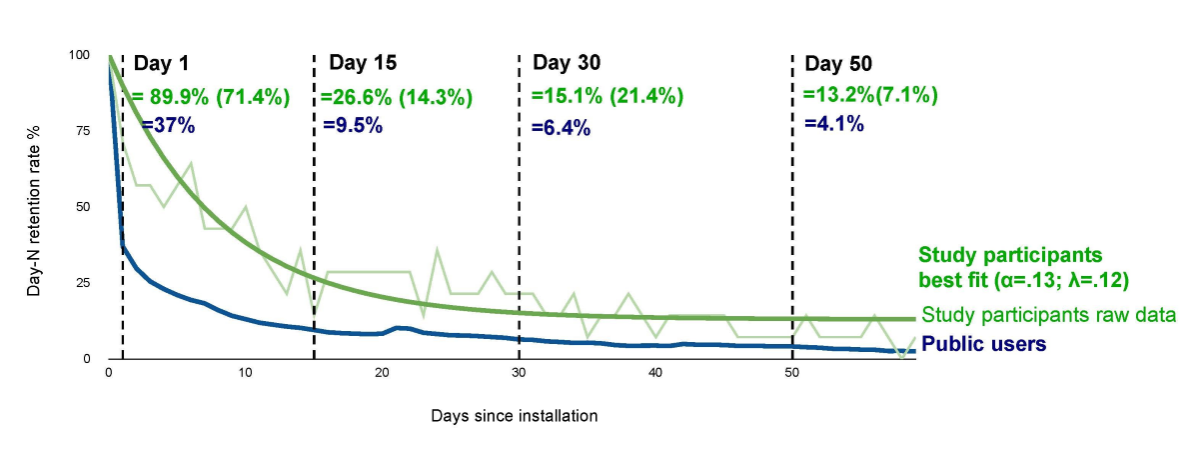
Day-N user retention. Day-N user retention rates from day 1 to day 50 for public users of The Guardians: Unite the Realms and the study participants, with both raw data and its best-fit line.

The average number of *activities completed* in *The Guardians* by participants (N=18) was 11.07 (SD 13.65), and the average number of *days played* was 16.67 (SD 17.52). The average activity completion rate (ie, rate at which a participant, on average, would complete an activity on any given day that they opened the gaming app) was 68.2% (SD 0.33). Of all the activities that study participants completed across the study period, 43.6% (65/149) were classified as *easy*, 27.5% (41/149) as *medium*, and 13.4% (20/149) as *hard* in effort level (the remaining were classified as *other* for effort level). Among the 56% (10/18) of the participants who completed MARS and PENS ratings at the final 10-week assessment, the average MARS rating was 3.49 (SD 0.76), and the average PENS rating was 4.13 (SD 1.66). All the correlations between engagement metrics (ie, days played and activities completed) and satisfaction measures (ie, MARS and PENS) were moderately positive (*r*=0.44-0.50) but not significant (with *P* values ranging from .13 to .20). Day-N study participant retention curves mapped on to those of the public *The Guardians* users are displayed in Figure 2. The optimal fit with all study data included is α=.13 and λ=0.12 with *R*^2^=0.93.

### Change in Clinical Symptoms

#### Overview

A series of independent 2-tailed *t* tests indicated no baseline differences in depression or anxiety scores between study completers (10/18, 56%) and noncompleters (8/18, 44%; *P*>.05). Table 2 summarizes the change in PHQ-9 and GAD-7 scores across the 10-week study.

**Figure 3:**
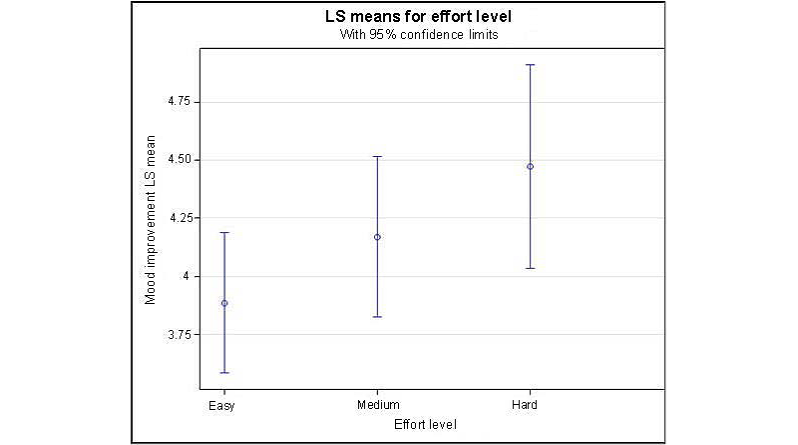
Means for postactivity mood improvement for various categories of activity effort level. Means are estimated via least squares (LS) regression and thus conventionally termed as “LS Means.”.

Regarding *postactivity mood improvement* ratings, participants reported feeling at least “a little better” 76% (113/149) of the time after completing an activity as part *of* *The Guardians* app. On average, participants reported having a greater improved mood after completing *hard* activities (mean 4.47, SD 0.21) compared with after completing activities identified as *medium* (mean 4.17, SD 0.16) or *easy* (mean 3.89, SD 0.14) in effort level. There was a significant (*P*=.05) relation between activity effort level and *post activity mood improvement*, with a gradually increasing mean *post activity mood improvement* rating from *Easy* to *Medium* to *Hard* effort levels of activities, as shown in Figure 3.

**Table 2:** Average scores on the 9-item Patient Health Questionnaire (PHQ-9) and Generalized Anxiety Disorder Scale-7 (GAD-7) across study assessment time points.

Measure	Baseline (N=18)	2 weeks (n=16)	4 weeks (n=12)	6 weeks (n=13)	8 weeks (n=12)	10 weeks (n=10)
PHQ-9 score, mean (SD)	13.17 (4.73)	9.88 (5.70)	8.67 (3.77)	9.92 (6.61)	9.58 (5.67)	7.2 (3.65)
GAD-7 score, mean (SD)	9.8 (4.18)	10.29 (5.62)	9.25 (4.03)	9.39 (5.46)	8.50 (3.50)	7.10 (4.12)

#### Change in PHQ-9 Score According to Time (Week in Study) and Activities Completed

For all the mixed effects models in the following sections, baseline age and gestational age were removed from the model as nonsignificant. For the first mixed effects model where the dependent variable was the PHQ-9 score, there was a significant negative main effect of linear *week in study* (unstandardized partial regression coefficient β=−.35; *t*_61_=−3.05; *P*=.003; 95% CI −0.59 to −0.12) and number of *activities completed* (β=−.12; *t*_61_=−2.05; *P*=.04; 95% CI −0.25 to −0.003). Overall, these fixed effect predictors accounted for 20.8% of the variance in the PHQ-9 score.

#### Change in PHQ-9 Score According to Time and Days Played

The same model as described previously was run except that number of *days played* replaced the number of *activities completed*. Results were analogous to those mentioned previously. There was a significant negative main effect of *days played* (β=−.12; *t*_58_=−2.9; *P*=.005; 95% CI −0.21 to −0.04) and linear *week in study* (β=−.34; *t*_58_=−2.86; *P*=.006; 95% CI −0.57 to −0.10). Overall, fixed effects accounted for 29.31% of the variance in the PHQ-9 score.

#### Change in GAD-7 Score According to Time and Activities Completed

**Figure 4:**
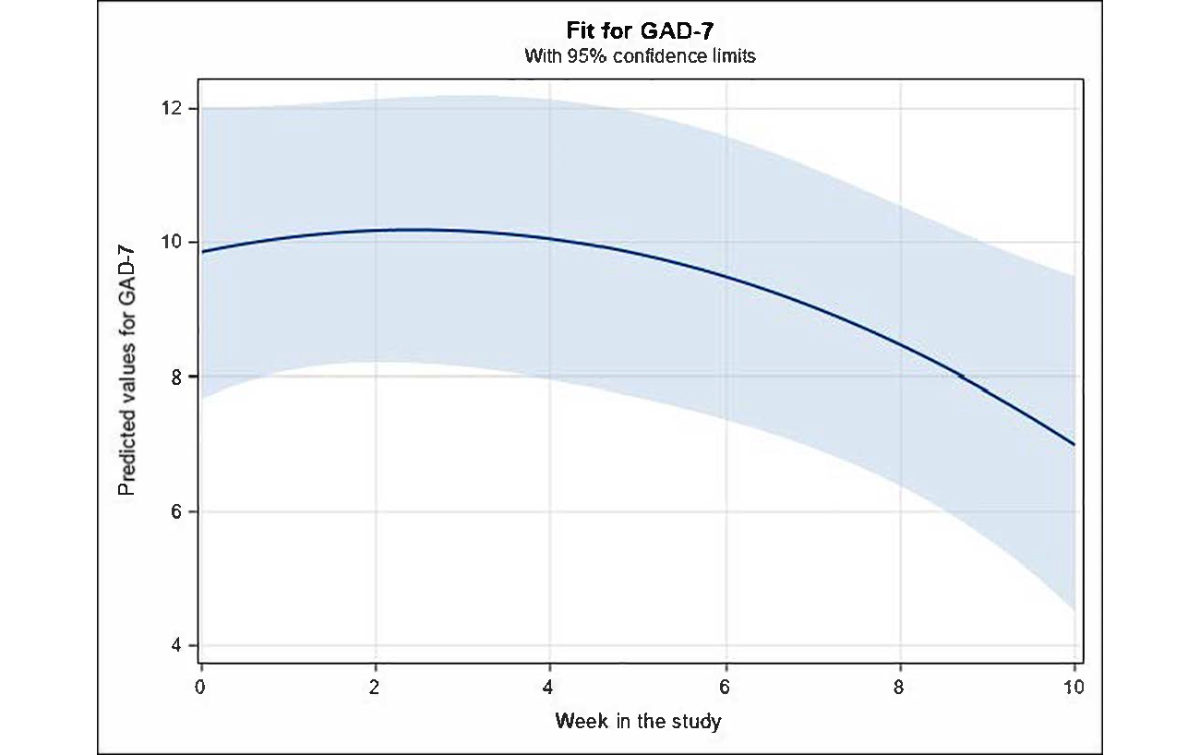
Mean values for Generalized Anxiety Disorder Scale-7 (GAD-7; with 95% CI) predicted by significant fixed effects in the fitted longitudinal model of GAD-7 versus week in study and activities completed. For fixed effects, a quadratic function for week in study was retained (marginally significant; *P*=.07), whereby the model–predicted GAD-7 values remained essentially stable up to approximately week 4.

The same model with the independent variables, *activities completed* and *week in study*, was rerun with GAD-7 score as the dependent variable. There were no significant effects of *activities completed* and only a marginally significant quadratic relation for *week in study* (β for linear term=.3; *P*=.38; β for quadratic term=−.06; *t*_45_=−1.89; *P*=.07; 95% CI −0.125 to 0.004). Overall, fixed effects accounted for only 4.2% of the variance in the GAD-7 score, whereas combined fixed *and* random effects accounted for 77.96% of the variance. Figure 4 displays the predicted values for this model and essentially indicates an accelerating decline in GAD-7 score across time, beginning at approximately week 4.

#### Change in GAD-7 Score According to Days Played

**Figure 5:**
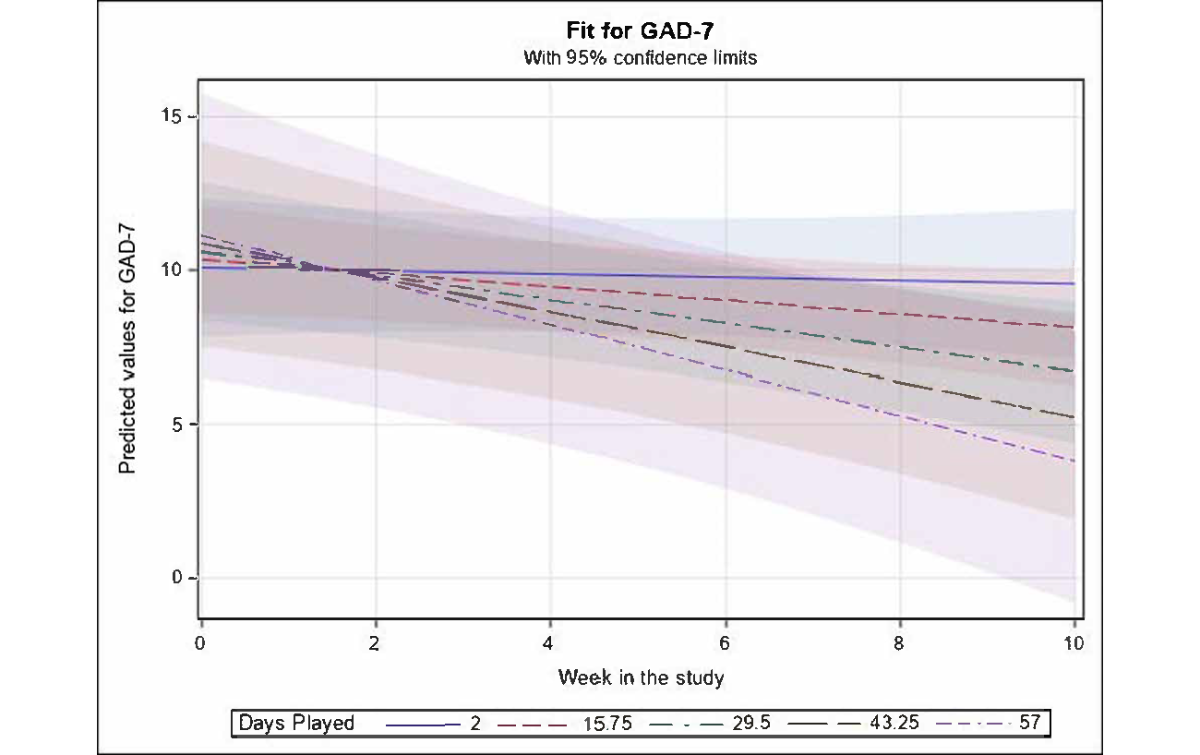
Mean values for Generalized Anxiety Disorder Scale-7 (GAD-7; with 95% CI) predicted by significant fixed effects in the fitted longitudinal model of GAD-7 versus week in study and days played. For fixed effects, only an interaction of days played × linear week in study was retained as significant (*P*=.05), whereby GAD-7 values were predicted by the model to remain essentially stable across time when the number of days played was relatively low (below 10) but showed an increasingly steep linear decline across time when the numbers of days played was increasingly high. Predicted values for GAD-7 are shown at selected illustrative strata of number of days played ranging from the minimum observed value of 2 up to the maximum observed value of 57 and at equally spaced intervals in between.

The same model as described previously was run with *days played* as a fixed effect substituted for *activities completed*. Although the main effect terms for linear *week in study* and for *days played* were not significant, there was a marginally significant interaction effect of linear *week in study* and *days played* (β=−.01; *t*_55_=−1.97; *P*=.05; 95% CI −0.02 to 0.0002), whereby GAD-7 score was predicted as essentially stable across time for participants with low numbers of *days played* but showed increasingly steep declines across time for those with increasingly high numbers of *days played* (Figure 5). Fixed effects accounted for 11.31% of the variance in the GAD-7 score.

#### Change in PHQ-9 Score Over Time According to MARS, PENS, and Treatment Started

**Figure 6:**
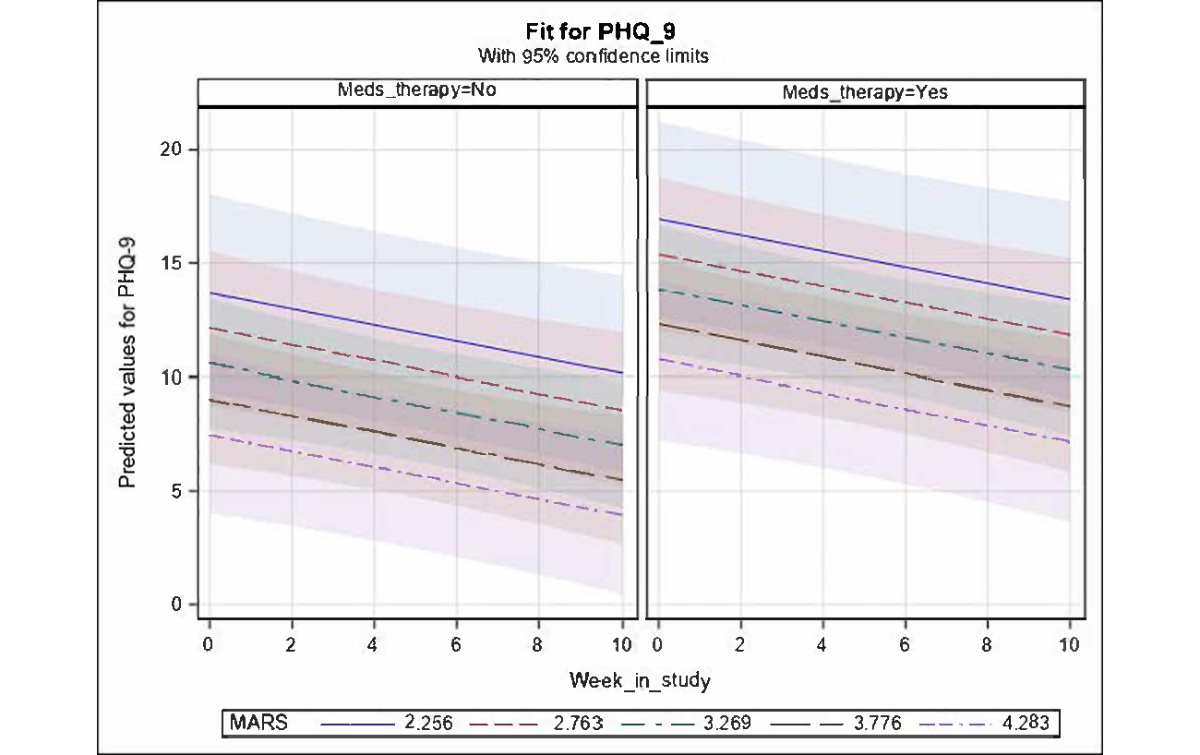
Mean values for Patient Health Questionnaire-9 (PHQ-9; with 95% CI) predicted by significant fixed effects in the fitted longitudinal model of PHQ-9 versus week in study, Mobile Application Rating Scale (MARS), Player Experience of Needs Satisfaction Scale, and treatment initiation (ie, whether medication or therapy was started or not started during the study). For fixed effects, only negative main effects for linear week in study (*P*=.009), MARS (*P*=.03), and treatment initiation (marginal; *P*=.07) were retained. Predicted values for PHQ-9 are shown in the respective panels for those who started or did not start treatment, across time within each panel and at selected illustrative strata of MARS ranging from the minimum observed value of 2.256 up to the maximum observed value of 4.283 and at equally spaced intervals in between.

In the model with MARS, PENS, *week in study*, and whether medication was started midstudy as independent variables and PHQ-9 score as the dependent variable, we found a significant negative main effect for linear *week in study* (β=−.35; *t*_45_=−2.74; *P*=.009; 95% CI −5.86 to −0.24) and MARS (β=−3.05; *t*_45_=−2.19; *P*=.03; Figure 6). The PENS variable was nonsignificant and was removed in the backward elimination. Additive to these effects, there was a marginally significant effect of treatment initiation (β=−3.25; *t*_45_=−1.84; *P*=.07), with the *treatment started* group (ie, medication or therapy started during the study period) estimated to have an adjusted mean of 11.45 (SD 1.2588), whereas the *no treatment started* group had a low adjusted mean of 8.20 (SD 1.2393). Overall, fixed effects accounted for 35.01% of the variance in the PHQ-9 score. There was no interaction effect between *treatment started* and reduction in PHQ-9 score across time; therefore, there was no significant difference in the slopes of change across time between the 2 medication groups.

#### Change in GAD-7 Score Over Time According to MARS, PENS, and Treatment Started

**Figure 7:**
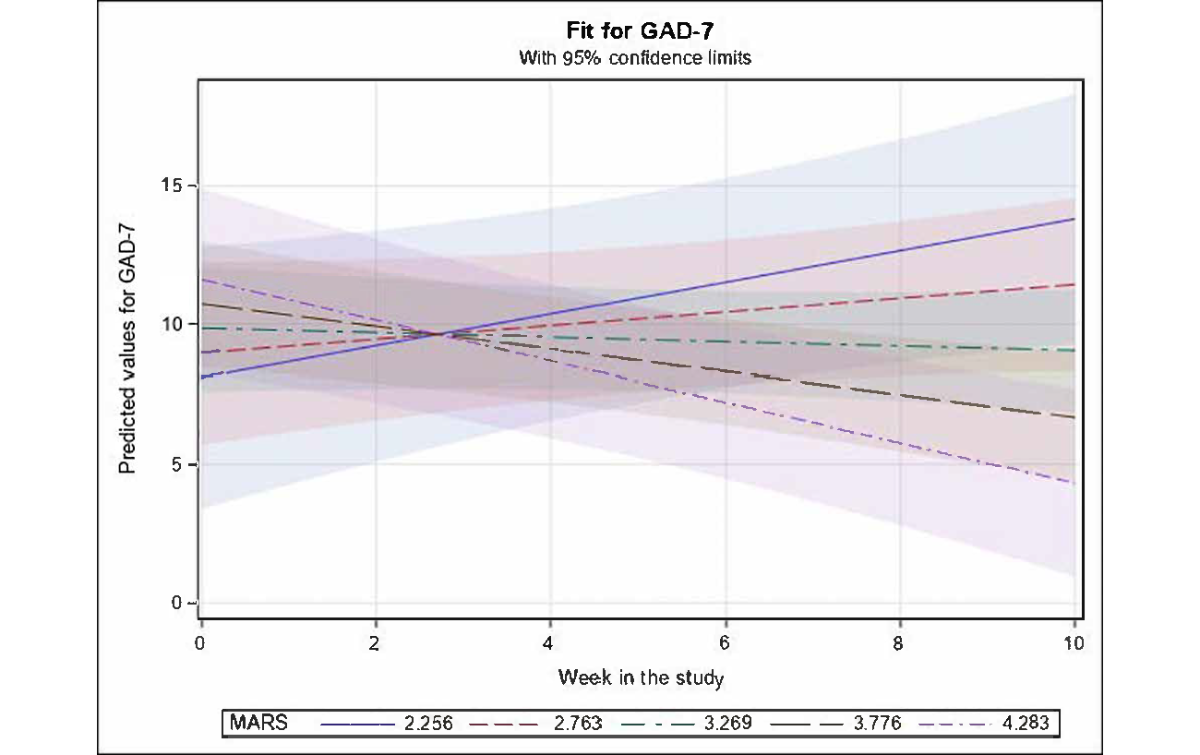
Mean values for Generalized Anxiety Disorder Scale-7 (GAD-7; with 95% CI) predicted by significant fixed effects in the fitted longitudinal model of GAD-7 versus week in study, Mobile Application Rating Scale (MARS), Player Experience of Needs Satisfaction Scale, and treatment initiation. For fixed effects, only an interaction of MARS × linear week in study was retained as significant (*P*=.002), whereby GAD-7 values were predicted by the model to vary from rising linearly across time among individuals with relatively lower MARS ratings to declining linearly across time for those with relatively higher MARS ratings. Selected illustrative strata of MARS in the figure were chosen to range from the minimum observed value of 2.256 up to the maximum observed value of 4.283 and at equally spaced intervals in between. The slight crossover of lines before approximately week 3 is likely just an artifact of the linear constraint for the lines and probably should not be interpreted with substantive meaning.

For the backward-eliminated mixed effects model for total GAD-7 score, there was a significant interaction between MARS and *weeks in study* (β=−.64; *t*_43_=−3.27; *P*=.002), reflecting the fact that as MARS ratings increased, GAD-7 score was predicted to decline more across time (Figure 7). PENS and *treatment started* variables were nonsignificant and removed in the backward elimination. Overall, fixed effects accounted for 18.07% of the variance in the GAD-7 score.

### Qualitative Feedback

#### Overview

Themes from the exit interviews fell into the following major categories: *app activities*, *app design*, *app engagement*, *fit of The Guardians with lifestyle*, and *perceived impact of The Guardians on mood*. Positive and negative feedback were identified in each of these categories. Participants also provided suggestions for app modifications, some of which were general recommendations and others were specific to pregnant app users.

#### App Activities

Participants reported that *The Guardians* encouraged completion of activities outside the game, as described in the following quote:

So, I would try to do daily house tasks...Like there was one, “clean-up a junk drawer,” and another one, like, “clean a room in your house.” So, I would try to pick those activities because, let’s face it, nobody ever wants to do those kinds of things. And that, I think, was kind of helpful for me, especially because I knew we were going to be moving, and it helped me declutter some stuff before we moved, so that was really awesome.

Participants also reported appreciating the variety of activities available. A participant shared the following:

I enjoyed the activities that were offered...there’s a wide variety of activities that would suit a lot of different people.

Some participants, however, had difficulty in completing certain activities (eg, “Doing longer [activities] was harder because at the time I was having some health issues myself...”). Others felt that it was easy to select activities that they were already doing in their daily lives, as described by a participant:

I mean, most of [the activities] were things that I was kind of doing already, like taking a shower, or I think, like, doing dishes...So honestly, I kind of picked the easy ones I was automatically doing.

#### App Design

Overall, participants appreciated the design of *The Guardians’* characters (eg, “The aesthetics were adorable; they’re really cute.”). A participant noted that the characters in the game motivated her to continue engaging with the app:

I think within the game itself, my biggest motivator was probably getting new pets. As someone who really likes arts and things like that, the designs of the creatures were really interesting. Being able to get a new creature each day was great.

Participants also appreciated the design of *The Guardians* itself, in terms of its esthetics (eg, “I did enjoy that the different levels had different world feels.”) and mechanics (eg, “...It’s one of the only apps that never crashed on me.”).

Some participants also had more critical feedback regarding the app’s design. For instance, a person felt that the app was difficult to navigate (eg, “If I didn’t feel like I was...fumbling my way through it, I could have maybe engaged faster and more.”). Others were frustrated that they had to wait before the next level unlocked (eg, “At one point, I think I had to wait, like, thirty days or something in order for the next level to open up, and I was like, ‘Oh, my goodness—what do I do now?’”). Similarly, some participants were frustrated by the length of the game’s introduction and overall complexity, as summarized by a woman:

I would say that the learning curve was just too much...like the mental state I’m in, plus, like, the amount that I’m trying to juggle. It was just a lot to try to add the additional learning of a game...

#### App Engagement

Participants shared that *The Guardians* allowed them to develop a sense of routine (eg, “When I was trying to get myself out of bed and stuff like that, [the app] just gave me another reason to get out of bed...”). They also enjoyed the gamification of daily household activities that may otherwise be difficult to complete while pregnant or experiencing elevated depressive symptoms (eg, “I definitely liked the aspect of...the little challenges of doing laundry and cleaning and stuff like that, because it kind of gamified doing that type of stuff.”). Furthermore, engagement with the app even helped a participant to start and maintain a healthy coping behavior beyond the end of the trial:

I think having that structure, having that reward to engage in a very common coping mechanism for me, really helped to motivate and get me in a pattern of reading that has continued, even if I’m not actively engaging with the app right now.

Not all participants, however, found *The Guardians* to be motivating or engaging (eg, “I didn’t really feel like [the app] was pushing me to come back in any way.”). Some found the app to be very complex, as previously described, whereas others felt that it was very simple (eg, “I think if it was a little bit more complex, it would have been more engaging for a longer period of time.”).

#### Fit of The Guardians Into Lifestyle

Some participants found that using *The Guardians* was an easy fit into their existing lifestyle (eg, “I would use [the app]...on the way to work.”). Furthermore, a participant felt that the game helped them return to a previous routine:

Once I started using the app in the mornings, I was able to get out of bed and do the things that I [had previously] been doing. So, it kind of snapped me back into my regular routine...

In terms of negative feedback, some participants felt that their busy schedules and lifestyles were not compatible with using *The Guardians* consistently. A participant stated the following:

I already have three kids, so I needed something to kind of be easy and mindless, and this was like...you had to be focused in on what you’re doing to even learn the game. So, for me, it was just too much.

#### Perceived Impact of The Guardians On Mood

Some participants reported that *The Guardians* positively influenced their mood (eg, “I think that the reward [was] my mood improving.”). Other participants did not find the app effective in influencing their mood (eg, when a participant was asked whether *The Guardians* affected their mood, the participant responded, “No, not really.”).

#### Suggestions for App Modifications

Participants had various recommendations for future improvements to *The Guardians*. Broadly, participants recommended simplifying the introduction to the app (eg, “Make it a little bit easier so it’s not so much upfront...to make sense of it.”). In addition, participants suggested a way to track activities in the game to monitor progress (eg, “I think if...it tracked [the activities], that would be really helpful, too.”).

Recommendations also centered around ideas for making *The Guardians* more relevant and engaging for pregnant individuals. Participants suggested adding more pregnancy-specific activities, as described in the following quote:

I think maybe for pregnant women specifically...if you made one of those activities, like, do your kick counts, or something...Just a reminder to some people, especially first-time moms, because they might not know to do those kinds of things...

Furthermore, some participants recognized that potential activities could differ based on gestational age. A participant shared the following:

For pregnant women, even having, like, a first trimester versus second trimester versus third trimester...There are different goals in there too, if you look at, like, pregnancy guidelines. Like, maybe, “Pick out something for the nursery today.” I think it could be interesting...to even look at how you can engage at different points of your trimesters.

Others recommended that *The Guardians* incorporate educational content related to pregnancy, as described in the following quote:

I think there are elements too, where there's pregnancy education that could be added into a gamification setting, where you are educating people at the same time as encouraging...healthy habits and things like that.

## Discussion

### Principal Findings

Our findings from this pilot study provide preliminary support for *The Guardians* as a potentially desirable intervention to engage and improve mood among some pregnant women. All enrolled participants met the criteria for a current MDE and the average baseline depressive symptoms fell into the *moderate* symptom severity level according to PHQ-9 [53], suggesting that *The Guardians* may be of interest to and able to engage pregnant individuals with moderate depressive symptom levels. Without providing any compensation for participation in the study, we enrolled 18 pregnant women within a brief, 8-month recruitment period, indicating that a gaming app such as *The Guardians* may be of interest to some individuals in the target pregnant population. Furthermore, one-third of the sample (6/18, 33.3%) identified as non-White, which is encouraging in that the app may be acceptable to a diverse population. However, we recognize that this was a small and relatively highly educated and employed sample; further studies are needed to assess the ability to engage a diverse perinatal population with elevated depressive symptoms using *The Guardians*.

Our user engagement data aligned with previous studies suggesting that *The Guardians* may be effective in improving long-term engagement relative to other digital mental health interventions [30]. The 15-day and 30-day overall app retention rates of 26.6% and 15.1%, respectively, compare favorably with the median retention rates of 3.9% (IQR 10.3%) and 3.3% (IQR 6.2%) for mental health apps, as reported by Baumel et al [27]. Furthermore, our retention rates suggest that *The Guardians* is favorably consistent with rates observed in the top 15% of entertainment-only mobile games that include engagement rates [54]. Such retention and engagement metrics are particularly encouraging, as there were no external (ie, outside the app) reminders for participants to use *The Guardians*, unlike other digital mental health interventions (eg, use of a therapist or coach to check in and encourage engagement). Although not statistically significant (likely owing to the small sample size in this study), it is not surprising that there were positive, moderate correlations between ratings of satisfaction with the app (as assessed using PENS and MARS) and engagement with the app (as assessed using days played and activities completed).

This was the first study to explore the change in depressive and anxiety symptoms among users of *The Guardians*. Our findings demonstrated reduction in both depressive and anxiety symptoms over the course of the 10-week study on average among study participants. Although we cannot attribute the decrease in clinical symptoms to the app owing to the lack of control group and this being an underpowered pilot study focused on feasibility, our analyses indicated statistically significant relationships between depression improvement and app engagement when there were less depression symptoms (according to PHQ-9), more activities were completed, and more *days played*. This offers some preliminary support that there may be improvement in depression when pregnant women engage more with *The Guardians*. Furthermore, low depressive symptoms were associated with high MARS rating of app satisfaction, indicating that there may be great improvement in depressive symptoms when a user is more satisfied with *The Guardians*. Overall, of the 2 app satisfaction ratings (ie, MARS and PENS), our mixed effects models found MARS to be a better predictor of change in clinical symptoms (main effect for decrease in PHQ-9 score; interaction with time for decrease in GAD-7 score) relative to PENS, which was removed in backward elimination for all of our analyses.

Our findings also suggest that there may be great improvement in mood after completing activities that require more effort. However, the effort level of app activities was assigned by the app developers, and activities determined to be *easy* or *hard* may not align with how a user would rate the effort for these activities. Further studies are needed to explore the relationship between the types and effort levels of activities completed and impact on mood. In addition, this study focused on individuals only during pregnancy and not during the postpartum period, as it seems there are unique aspects of the postpartum period (eg, sleep deprivation and demands of a newborn) that may influence the ability to engage with the app or complete certain activities. Future studies could assess the impact of this app in the postpartum period and further explore how certain activities in the postpartum period are perceived in terms of effort level and impact on mood.

Our findings regarding change in anxiety symptoms revealed an interaction between *days played* and *time*, indicating that there were relatively stable anxiety levels on average among participants who used the app less, but there was great improvement in anxiety scores for those who used the app more (ie, great number of days played). This provides some support for the potential benefit of using *The Guardians* among this sample of pregnant individuals with anxiety and elevated depression, yet only for those who used the app more often. Not surprisingly, given the nonsignificant but positive correlation between app satisfaction (ie, MARS) and *days played*, there was great improvement in anxiety symptoms when the MARS ratings were high (ie, high acceptability). Again, further studies are needed with a powered RCT to explore these changes in clinical symptoms among users of *The Guardians*, including assessment of other factors that may be contributing to change in clinical symptoms (eg, specifics about treatment changes during the study and major life events). Our findings indicate that participants who started treatment (medication or psychotherapy) during the course of the trial had great depressive symptoms both at baseline and at the end of the 10-week trial; however, the rate of change in depressive symptoms between these groups over the course of the study period was similar.

The qualitative feedback provided by participants indicated that there were contrasting opinions about certain aspects of the app. Participants tended to positively assess the activities on the app if they were novel to the participant or suited their individual lifestyles and abilities. Participants tended to negatively assess the activities if they felt that they were very difficult or did not have a significant or positive effect on their mood. There were also some contrasting views about the nature of the app; some felt that it was “mindless” and fun, whereas others felt that it was very demanding and time consuming. Future studies could explore ways to tailor the app to make it more or less challenging, depending on a person’s preferences or needs. On the basis of participants’ recommendations, other features could be added in future iterations of the app, such as an activity tracking module to help monitor activity completion and impact on mood over time. Other feedback from participants will be critical in informing the adaptation of *The Guardians* to a perinatal population to include pregnancy-specific activities and educational content. It is possible that a more tailored version of the app for perinatal individuals with depression may be even more feasible, acceptable, and effective in reducing depressive symptoms for this population.

### Limitations

This study has several limitations. As indicated previously, the study sample was small and relatively highly educated and employed. Thus, we do not know how generalizable these findings are to a broad population. As this pilot study did not have a control condition, we cannot make any causal claims regarding the impact of the app on clinical symptoms. Furthermore, future studies should assess how access to smartphones or comfort with technology may influence who would engage with an app such as *The Guardians.* A large RCT is needed to further explore how *The Guardians* may be used among pregnant individuals across socioeconomic levels and to rigorously assess the app’s effectiveness in treating PD.

### Conclusions

*The Guardians* is a widely available and highly engaging gaming app that incorporates BA principles. In this study, we sought to explore the usability, acceptability, and preliminary effectiveness of this app among a small sample of pregnant individuals with elevated depressive symptoms. Findings from this small pilot study provide initial support for *The Guardians* as an acceptable and engaging app, and there may be some improvement in mood and anxiety among certain users in the target population. This was one of the first longitudinal pilot studies to explore the effectiveness of a BA gaming app on PD. Further studies, including a powered RCT, is needed to follow-up on these preliminary findings.

## Appendix

Multimedia Appendix 1 Exit interview questions.

## Abbreviations

BA: behavioral activation
GAD-7: Generalized Anxiety Disorder-7
MARS: Mobile Application Rating Scale
MDE: major depressive episode
PD: perinatal depression
PENS: Player Experience of Needs Satisfaction scale
PHQ-9: Patient Health Questionnaire-9
RCT: randomized controlled trial
REDCap: Research Electronic Data Capture

## Data Availability

The data sets generated and analyzed during this study are available from the corresponding author upon reasonable request.
