# Supplementary material for: Testing a Behavioral Activation Gaming App for Depression During Pregnancy: Multimethod Pilot Study
Source: JMIR Form Res. 2024 Jan 26;8:e44029. doi: 10.2196/44029 (PMC10858420; doi:10.2196/44029)
Supplement: Multimedia Appendix 1 [file formative_v8i1e44029_app1.docx]

Multimedia Appendix 1

Exit Interview Questions

- Please describe your level of engagement with the app since the start of this study. Probe for:
  - How often did you log in?
  - When would you use the app?
    - Do you feel that using the app became a part of your routine (daily or weekly)?
    - What prompted you to use the app? Were the app’s notifications a reminder to use it, or did other cues prompt you to play the game? Did this change over time?
    - Did you have preferred times of day or days of the week to use the app?
    - Did you have preferred times of day or days of the week to complete your daily adventure?
  - When you used the app, how long would you use it for?
  - How often did you complete the real-world activities?
- What did you like about the app?
  - *Probe for content, design, aesthetics...*
- What did you not like about the app?
  - *Probe for content, design, aesthetics...*
    - Do you have any feedback on the plot?
    - Do you have any feedback on the art design?
    - Do you have any feedback on the gameplay features (e.g., completing missions)?
    - Do you have any pets you particularly liked or disliked?
    - Were any of the features confusing?
- Please describe how effective you found the app at helping you to manage your mental health. Probe for:
  - How did the app impact your mood, if at all? Please describe
  - Could you find activities that you enjoyed and found effective?
  - Do you have any suggestions for new daily activities / adventures?
  - Do you have any suggestions on how to improve the experience of finding, completing, and reflecting on daily activities?
    - Did the app help you to learn which activities help to improve your mood?
    - Would you like to see more data on your history of completing activities and rating them?
    - Would the ability to schedule activities in the future be helpful?
  - Did you find it motivating to receive rewards for your real-world activities?
    - Do you have any suggestions on how to improve the experience of receiving rewards?
  - Were there any factors in the game that you found demotivating?
- What suggestions do you have for how to make the app more engaging for pregnant women?
- What other features would you add or change with this app to support women who are experiencing depressive symptoms in pregnancy?
